# Evolving Castration Resistance and Prostate Specific Membrane Antigen Expression: Implications for Patient Management

**DOI:** 10.3390/cancers13143556

**Published:** 2021-07-16

**Authors:** Katharina Kessel, Christof Bernemann, Martin Bögemann, Kambiz Rahbar

**Affiliations:** 1 Department of Nuclear Medicine, University Hospital Münster (UKM), 48149 Münster, Germany; kambiz.rahbar@ukmuenster.de; 2 Translational Tumor Biology Section, Department of Urology and Pediatric Urology, University Hospital Münster (UKM), 48149 Münster, Germany; christof.bernemann@ukmuenster.de; 3 Department of Urology and Pediatric Urology, University Hospital Münster (UKM), 48149 Münster, Germany; Martin.Boegemann@ukmuenster.de; 4 Network Partner Site Westdeutsches Tumorzentrum (WTZ), 45147 Essen, Germany

**Keywords:** prostate-specific membrane antigen, PSMA, metastatic castration-resistant prostate cancer, mCRPC, ^177^Lu-PSMA-RLT, theranostics

## Abstract

**Simple Summary:**

This review is a summary of recent findings on the role of prostate-specific membrane antigen (PSMA) in metastatic castration-resistant prostate cancer (mCRPC) and how they can be implemented into patient management. The multiple aspects, interactions and functions of PSMA expression should be considered with regard to diagnosis and treatment. Approval of ^177^Lu-PSMA radioligand therapy (PSMA-RLT) by the FDA is impending and might lead to broader indications for application than third-line therapy of mCRPC. Earlier use of PSMA-RLT and combinatorial approaches with other novel agents are the promising future of mCRPC management.

**Abstract:**

Metastatic castration-resistant prostate cancer (mCRPC) remains an incurable disease, despite multiple novel treatment options. The role of prostate-specific membrane antigen (PSMA) in the process of mCRPC development has long been underestimated. During the last years, a new understanding of the underlying molecular mechanisms of rising PSMA expression and its association with disease progression has emerged. Accurate understanding of these complex interactions is indispensable for a precise diagnostic process and ultimately successful treatment of advanced prostate cancer. The combination of different novel therapeutics such as androgen deprivation agents, 177LU-PSMA radioligand therapy and PARP inhibitors promises a new kind of efficacy. In this review, we summarize the current knowledge about the most relevant molecular mechanisms around PSMA in mCRPC development and how they can be implemented in mCRPC management.

## 1. Introduction

Prostate cancer (PCA) is the most common male cancer with approximately 60,000 new cases in Germany each year [1]. PCA represents the most frequent cause of cancer-related deaths in men. With an average age of 65 at initial diagnosis, PCA is a disease of ageing men with age as one of the major risk factors. Further risk factors are unknown, while lifestyle and diet have been discussed to play a potential role in disease etiology. Due to ageing populations in Germany (Federal Statistical Office https://www.bib.bund.de, accessed on 8 July 2021) and worldwide (American Cancer Society: https://www.cancer.org/cancer/prostate-cancer/about/key-statistics.html, accessed on 8 July 2021), quick and safe diagnosis and treatment of age-related diseases become highly relevant and essential. Increasing the understanding of the underlying molecular mechanisms of PCA stages and their progression is therefore crucial for optimal and patient-specific treatments.

Most patients recover after prostatectomy after initial diagnosis. However, about 20% present with advanced disease or metastases at initial presentation [2]. About one third of patients develop recurrent disease after prostatectomy or definitive radiotherapy [3,4]. Androgen deprivation therapy (ADT) is the usually the first line of treatment for those cases [5]. In general, this therapy is successful in the first place for androgen-dependent tumors. Yet, 10–20% of patients become resistant to ADT and enter the stage of castration resistance (metastatic castration-resistant prostate cancer: mCRPC) [6]. First-line treatment for those patients is the next-generation CYP17A1 inhibitor abiraterone acetate or inhibitors of the androgen receptor pathway such as enzalutamide, as well as apalutamide and darolutamide representing the latest treatment options [7,8,9,10]. Chemotherapy with docetaxel or cabazitaxel are the options to choose from in case of ADT failure [10,11,12,13]. One measurable feature of ADT is the rise of prostate-specific membrane antigen (PSMA) expression in the tumor and its metastases, which can be used for both therapy and diagnostics [14]. Therefore, PSMA represents the ideal theranostic target in PCA and might serve as a safe prognostic biomarker, as well as a powerful treatment option. Additionally, PSMA seems to play a major role in the progression to castration resistance, which is potentially induced or promoted by androgen deprivation therapy (ADT) [15].

The advent of PSMA-targeted radioligand therapy (PSMA-RLT) for advanced mCRPC almost ten years ago has clearly enriched therapy options to choose from with regard to mCRPC treatment for doctors worldwide [14]. Ever since its first therapeutic application in men, it has proven safety and efficiency [16]. Currently, PSMA-RLT is recommended and used as a third line of treatment only after failure of ADT and chemotherapy as an alternative healing attempt. It has been shown that PSMA-RLT is superior in third line compared to chemotherapy with cabazitaxel and androgen receptor (AR)-signaling inhibition [17]. In the last few years, data on PSMA-RLT have increasingly been collected and the outcome supports its use [18]. After the phase III VISION trial, PSMA-RLT approval is impending and therefore it is time to discuss and evaluate its temporal application during treatment sequences in prostate cancer [19,20]. Patients worldwide might benefit from a PSMA-RLT that can be applied by physicians more flexibl and also at earlier stages, as well as in combination with other potent therapies.

^68^Ga-PSMA positron-emission topography (PSMA-PET) allows PSMA detection with high precision and low toxicity, making PSMA the ideal theranostic target. Rising PSMA during evolving castration resistance and a positive correlation with disease progression adds to this favorable biomarker profile of PSMA [21,22,23]. However, although there is a reported relationship between tumor burden and PSMA expression, it does not always display a positive correlation, especially under ADT. Tumor load and PSMA expression intensity measured by PSMA-PET therefore have to be considered two different aspects of disease severity during diagnostics [15].

Little was known about the biological function of PSMA in cancer, nor about its role in the progression of prostate cancer into the stage of castration resistance and eventually neuroendocrine PCA, until very recently. Slowly, we begin to fully understand the role of PSMA in recurrent and progressing prostate cancer and its impact on evolving castration resistance in more detail. In this review, we will dissect the role of PSMA in evolving castration resistance and why this makes it not only a valuable therapeutic target in late mCRPC, but also a valid and safe biomarker for imaging and disease staging [24].

## 2. Castration Resistance

PCA develops from a prostatic intraepithelial neoplasia (PIN) as an asymptomatic precursor of adenocarcinoma. Through accumulation of progressive somatic mutations and loss of distinct basal and secretory cell layers, PIN eventually evolves into a local prostate tumor, bearing the potential to metastasize through local invasion or lymphatic and hematogenic pathways. Rising prostate-specific antigen (PSA) serum levels offer the possibility to detect PCA early; however, the PSA blood test does not allow discrimination between low- and high-risk tumors [25]. Therefore, standard of care for the diagnosis of PCA is the microscopic evaluation of a prostate tissue biopsy [26]. Gleason grading of the samples is then performed by a pathologist, which allows stratification into low, intermediate and high risk. Initial therapy of localized PCA without any involvement of lymph nodes or distant metastases consists of surgical removal of the prostate and affected tissue and/or radiation therapy. In some cases, watchful waiting and active surveillance involving serial PSA testing, physical examinations and biopsies can be performed [27]. Hormone responsiveness to androgens increases PCA aggressiveness, which is why androgen deprivation therapy (ADT) is now the standard of care for hormone-sensitive prostate cancer [10,28]. As a first-line treatment, ADT with LHRH analogs is widely used for non-metastatic and metastatic PCA [29]. ADT non-responders are often treated with chemotherapeutics such as docetaxel or cabazitaxel or a combination of ADT and chemotherapy [28]. In many cases, metastatic PCA becomes unresponsive to ADT, and is thereby classified as metastatic castration-resistant prostate cancer (mCRPC), which is often accompanied by a rise in PSMA expression [30,31]. Currently, cytotoxic agents, androgen receptor targeting, immunotherapies and radiopharmaceuticals are available for the treatment of mCRPC [32]. The standard treatment sequence involves taxane chemotherapeutics (docetaxel and cabazitaxel) and novel androgen deprivation therapy (abiraterone acetate, enzalutamide, apalutamide and darolutamide). Sipuleucel-T, an immune-modulatory cellular therapy, is only approved in the US, and treatment with Radium-223 for bone metastases has been moved to the third line of treatment due to a restriction of use by the EMA, although it is of benefit for particular patients [33,34]. First-line treatment of mCRPC is usually performed with abiraterone acetate and prednisone, enzalutamide, docetaxel or cabazitaxel. Second-line treatment consists of enzalutamide and docetaxel, and in rare cases, Radium-223 [10]. Although effective in those late stages of PCA, enzalutamide is also approved for first-line treatment in hormone-sensitive prostate cancer in combination with androgen deprivation [35,36]. Despite a number of possible treatments, mCRPC is considered incurable, with a median OS of 18–30 months.

There is a reported relationship with 17–30% incidence between advanced PCAs, which exhibit classical and next-generation ADT resistance, and downstream neuroendocrine-like differentiation (NELD) [37]. Rising serum PSA despite ADT is a possible key feature of mCRPC and calls for novel diagnostic and therapeutic strategies to earlier and more accurately identify mCRPC. Besides metastases, expression of androgen receptor (AR) and its variants despite therapeutic suppression of the AR axis classifies mCRPC. Additionally, changes in PSMA expression levels might also be taken into consideration for disease monitoring other than for favorable imaging conditions. Even more so, as there is an increasing understanding about a role for PSMA in the activation of the IP3K–AKT–mTOR cascade [38]. Loss of PTEN expression [39], BRCA1/2 mutations [40] and PARP overexpression [41] occur with progressing disease and may be used for molecular sub-staging of mCRPC. Newly introduced treatment with olaparib, a PARP inhibitor, revealed promising success in BRCA1/2-mutated and non-mutated settings [42,43]. PARP-2 overexpression further was shown to restore AR signaling via transcription factor FOXA1 disruption [44]. Targeting mechanisms of DNA repair seems to have the greatest impact of tumor growth in those late stages of mCRPC, which is why this is increasingly investigated among the latest literature [43].

Although we are provided multiple modern state-of-the-art screening technologies and opportunities for molecular testing, these are rarely used to tightly monitor ongoing disease during mCRPC patient management. There is reason to use biopsies; however, the effort and violation of the patient to receive the material would need more justification if it was to be used for global genomic and gene expression profiling [45]. Additionally, physicians worldwide should make more use of liquid biopsies, to include more data for predicting therapeutic efficiencies or arising resistance mechanisms [46]. With focus on PSMA as a theranostic target and a potent biomarker of tissue samples and imaging, mCRPC development could be monitored more precisely [24,47]. These data urgently require consideration and deserve more attention in clinical decision making, to build the basis for molecular targeting of tumor cells. Further, such profiling would allow us to generate a map of each individual patient and the active pathways that determine the disease stage and risk of progression. All this would truly allow the much-conjured patient-specific precision medicine [45].

## 3. PSMA Biology

### 3.1. The Multiple Functions of PSMA

PSMA was originally discovered as a folate folypoly glutamate carboxypeptidase (FGCP) and was found in healthy jejunal brush border membrane, prostate, proximal tubules of the kidney and nervous system glia, as well as salivary glands [48,49,50]. However, prostate tissue seems to be the only site with significant expression [50,51]. Further, PSMA is found in malign conditions such as prostate and breast cancer, as well as in the neovasculature of a variety of tumors, including renal cell carcinoma, but also in regeneration and repair in various tissues [52,53]. Because of its strong expression in prostate tissue, the protein is mostly named prostate-specific membrane antigen—PSMA. Encoded by the gene FOLH1, this enzyme has peptidase [54] and hydrolase [55] activity and digests dietary polyglutamylated to monoglutamylated folates [56,57]. While low serum folate is associated with a number of other cancers, increased serum levels and supplementation may be associated with a higher risk of prostate cancer, although this is debatable based on the current literature [58,59]. Intracellular folic acid (Vitamin B9) concentration itself has been shown to provide a proliferative advantage to PSMA-expressing LNCap cells in vitro. Therefore, elevated PSMA expression might aid with tumor cell proliferation in PCA, when supplied with excess folate [60]. Correspondingly, it has been shown that PSMA-expressing tumors in LNCaP mouse xenografts grow larger in response to dietary folate [61]. This indicates that patients with PSMA-expressing tumors might reduce folate intake and adjust their diet accordingly to support specific treatments [62]. PSMA, besides carrying the description FGCP, was also detected as N-acetyated-alpha-linked-acidic dipeptidase 1 (NAALAD1) in the nervous system [63]. Its increased expression or activity might be responsible for excitotoxicity in numerous neurological diseases, such as multiple sclerosis [64]. With regard to prostate tissue and renal tubule, the role of PSMA expression is not yet well defined. Most likely, PSMA functions as a receptor for folate re-uptake (kidney) or helps to release monoglutamated folate into the seminal fluid, where it influences sperm density and count [65]. The availability of folate can increase the level of nitric oxide which has proangiogenic functions by regenerating the endothelial nitric oxidase synthase (sNOS) cofactor tetrahydrobiopterin (BH_4_), which is known to help with angiogenesis, thus making PSMA an interesting target in antiangiogenic scenarios, which has been shown to be integrin-mediated [52,53,66,67]. Further, expression of PSMA has been shown to increase from benign prostate hyperplasia (BPH) to PCA and to correlate with Gleason score and disease aggressiveness, which implies a role for PSMA not only in proliferation, but also in tumor malignity [51,68,69,70]. Very recently, multiple complex molecular interactions of PSMA during evolving castration resistance were reported, and revealed a promoting function of PSMA in the most relevant signaling pathways of cancer cell aggressiveness.

### 3.2. PSMA Internalization and Its Relevance to Theranostics

PSMA expression is associated with early serum PSA recurrence and disease progression. Therefore, it can be considered a valid biomarker for disease monitoring [71]. Further, PSMA seems to have a receptor–transporter function, as ligands such as antibodies and small molecules can be internalized [57]. However, even if the protein with its enzymatic functions is well characterized, there is no known natural ligand to PSMA to date, although there have been countless studies which attempted to define the optimal binding motif [72,73,74,75]. Regardless of this lack in knowledge, its internalization is effectively used in PSMA radioligand therapy (PSMA-RLT), where a radio-labeled ligand travels into the cell via PSMA internalization and induces double-strand breaks through local radiation [76], as well as in PSMA PET imaging.

**Figure 1:**
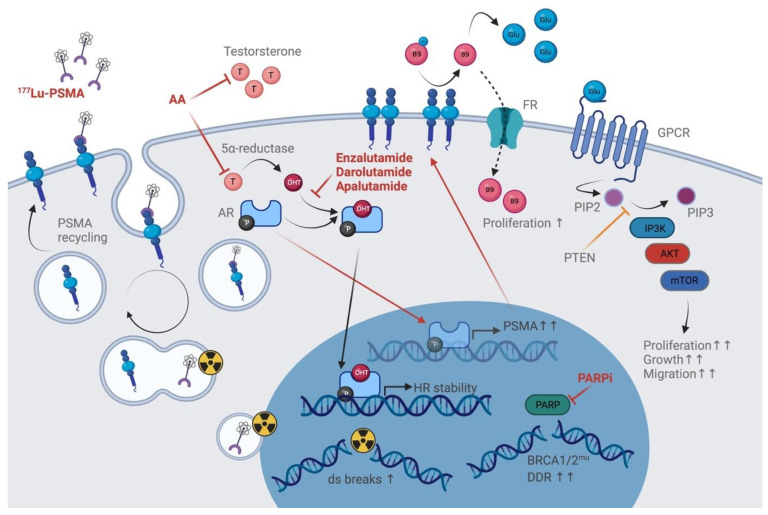
Multiple molecular interactions in prostate cancer cells. PSMA is internalized upon ligand binding, in this case the radiolabeled small molecule ^177^Lu-PSMA-617. The ligand is transported to the perinuclear region for degradation and releases its β-radiation to induce ds breaks. The PSMA molecule is recycled and released to the cell surface. Testosterone binds to the AR in the cytoplasm. The AR–testosterone complex binds to binding motifs on the DNA and ensures HR. If suppressed by AA or enzalutamide, unbound AR binds to the PSMA enhancer and promotor region and upregulates PSMA expression. PSMA has enzymatic properties and releases free glutamate from polyglutamated folate (B9). B9 is transported into the cell via the FR and aids with proliferation. Free glutamates activate GPCRs, which downstream activate IP3K if not inhibited by PTEN. Via IP3K-mediated conversion of PIP2 to PIP3, activated IP3K–AKT–mTOR signaling promotes proliferation and tumor cell growth. In BRCA1/2-mutated cells, PARP is upregulated and strongly induces DDR. PARPi counteracts PARP-mediated DDR. Black arrows and grey letters indicate normal function; red arrows and letters indicate drug-mediated functions. 177LU: 177Lutetium; PSMA: prostate-specific membrane antigen; AA: abiraterone actetate; DHT: dihydroxy-testosterone; T: testosterone; HR: homologous recombination; ds: double-strand; Glu: glutamate; FR: folate receptor; GPCR: G-protein-coupled receptor; PIP2: phosphatidylinositol-4, 5-bisphosphate; PIP3: phosphatidylinositol-3, 4, 5-triphosphate; IP3K: inositol 1,4,5-trisphosphate 3-kinase; AKT: protein kinase B; mTOR: mammalian target of rapamycin; PTEN: phosphatase and tensin homolog on chromosome ten; PARP: poly(ADP-ribose)-polymerase; PARPi: PARP inhibitor; BRCA1/2mu: breast cancer 1/2 mutation; HR: homologous recombination; DDR: DNA-damage repair. This figure was generated using biorender, www.biorender.com, accessed on 8 July 2021.

After binding and internalization, the receptor is recycled, as other membrane-bound receptors, via clathrin-coated pits and association with filamin A [77,78,79]. During this process, the ligand-bound PSMA receptor is internalized to the sorting endosome [57]. In the following, the receptor–ligand is either degraded in lysosomes, or free receptors are recycled back to the cell membrane. Whether PSMA exclusively accumulates in the recycling endosomal compartment has not been finally proven, but there is no proof for accumulation in the perinuclear region of the cell which commonly leads to degradation [78]. In vitro studies and in silico modeling approaches showed that ligand-bound receptor internalization was about five times greater than for unbound receptors, which is very similar to other ligand–receptor internalization systems [80]. Internalization rates of antibody-bound PSMA have been described as 60% within 20 min at saturation, compared to two hours for 60% unbound PSMA [57]. Internalization rates are influenced by association and dissociation rates of the ligand and efficiency of therapy depends on ligand activity, while increasing ligand amount does not seem to increase uptake. According to in silico studies, currently used ligands seem to fulfill all prerequisites for optimal amount, binding and internalization [81,82]. Therapy efficiency and improved imaging can therefore only be influenced by radionuclide nature and activity, while ligand amounts are maintained [81]. The beta radionuclide ^177^Lu has been proven to be the safest radionuclide compared to ^225^Ac in the treatment of mCRPC, and ^68^Ga is mostly used for imaging [83,84]. Nonetheless, elevated PSMA through PCA progress or due to androgen deprivation builds the optimal target for PSMA-RLT. For a comprehensive and schematic overview of PSMA internalization, please refer to Figure 1.

### 3.3. The Androgen Receptor PSMA Axis

PSMA expression is inversely modulated by androgens and the androgen receptor (AR) signaling cascade [15]. Normally, 5^α^-dihydroxy-testosterone travels into the cell, is converted to testosterone by the 5^α^-reductase and binds to the AR. The AR–testosterone complex then binds to its target DNA motif in the nucleus. PSMA expression seems to be suppressed by the AR–testosterone complex, while it is upregulated by unbound AR. Expression of the PSMA-encoding gene FOLH1 is downregulated by androgens which bind the androgen receptor. Therefore, ADT conversely stimulates FOLH1 transcription and translation by non-androgen-bound AR [15]. The FOLH1 gene exhibits multiple binding sites for AR in its promoter and enhancer and can be considered a target gene of the AR signaling cascade [15,85,86]. However, it has to be mentioned that information from the literature regarding this interplay between PSMA and AR and its effect on tumor growth is heterogeneous, and we have to conclude that the mechanism is not yet fully understood [15].

Upon androgen deprivation therapy or AR blockade, the PSMA level rises in the tumor tissue and its metastases at the cellular level, thus enabling therapeutic targeting as well as detection by PSMA-based imaging. As an effect of efficient biochemical castration through ADT, however, the overall tumor size may decrease after castration or enzalutamide treatment [87]. After ADT halt, PSMA levels may eventually decrease to baseline within a few days, up to one week [88]. However, elevated PSMA expression was also shown to positively correlate with faster biochemical recurrence and metastasis [38]. Even more so, as PSMA was shown to be associated with the activation of the PI3K–AKT–mTOR oncogenic signaling pathway in a phosphatase and tensin homolog tumor suppressor on chromosome 10 (PTEN)-independent manner by Kaittanis et al. very recently [38]. Its proangiogenic properties might also aid tumor growth and invasion [52]. Further, Kaittanis et al. showed that PSMA induces elevated intracellular Ca^2+^ levels via IP3. Ca^2+^ aids proliferation, migration and vascularization in general, but also activates mTORC2 and thus promotes the whole process of tumor growth and proliferation, which is a hallmark of cancer cells [38,89,90]. Mammalian target of rapamycin (mTOR)-controlled autophagy is also affected by intracellular Ca^2+^ levels and might support cancer cell survival strategies [91]. Therefore, theranostic induction of PSMA by ADT is a double-edged sword, which has to be carefully considered with regard to stage-related temporal application. The majority of clinical studies describe an increase in PSMA expression by short-term ADT, but which may be downregulated during long-term treatment [15,92]. Androgen-resistant PCA then often presents with PSMA overexpression, while neuroendocrine PCA is accompanied by PSMA suppression [68]. However, although AR signaling is best known for its regulation of PSMA expression with clinical relevance, the underlying molecular mechanisms are only incompletely understood. Androgen deprivation therapy (ADT) is the standard of care for patients progressing after initial therapy and/or prostatectomy. Both inhibition of androgen synthesis by abiraterone acetate or androgen receptor (AR) inhibition by enzalutamide increase PSMA expression [15,93,94]. This effect has been shown to even function in enzalutamide-refractory cells via PSMA-PET imaging and is reversible, as previous PSMA levels are restored after halt [88]. Although rising PSMA provides a target for more therapy options, it might also contribute to androgen-independent disease progression [95]. Its described role in building tumor neovasculature and promoting proliferation may forward disease progression and the development of castration resistance and maybe downstream neuroendocrine differentiation (NED). A schematic of the AR–PSMA axis can be found in Figure 1.

### 3.4. PSMA, a Global Player in Prostate Cancer Progression

Treatment of the AR pathway has long been and is still the treatment of choice for refractory metastatic CRPC. Very recently, however, it was demonstrated by mRNA transcriptome analyses that AR inhibition activates the PI3K pathway [96,97]. In the respective study, Carver and his colleagues additionally showed that PI3K pathway interruption also restored AR signaling, even with a PTEN-deficient background. PTEN acts as a lipid phosphatase and converts PIP_3_ to PIP_2_, and thereby antagonizes the function of PI3K. Through this mechanism, PTEN suppresses the activation of the downstream oncogenic RAC-alpha serine/threonine-protein kinase (AKT) and mammalian target of rapamycin (mTOR) signaling pathway. Further, PTEN provides centromere stability and double-strand break repair [39]. PTEN loss is a feature of advanced PCA and has been reported to be associated with progression and worse disease outcome, especially when detected in isolated circulating tumor cells (CTCs) [98]. Partial or complete PTEN loss is acquired by genomic deletion during PCA progression and thus its inhibitory function on IP3K and activation of DNA ds repair is removed from the cellular functional self-regulatory network [39]. This loss of function of PTEN might be particularly crucial in PSMA overexpressing cells and lead to even worse prognosis. Based on the reported function and activating effect of PSMA on the IP3K–AKT–mTOR pathway, a PTEN loss might even more strongly allow cancer cells to proliferate, migrate and escape, given control mechanisms [38].

Enzymatic cleavage of glutamate from poly-glutamated folate by PSMA increases the level of free glutamates, which potentially bind to and activate a G-protein coupled receptor (GPCR). The GPCR in turn then activates PI3K and downstream AKT and mTOR, leading to increased cell growth and proliferation in cancer cells [99]. As if this were not enough, one study showed that PSMA activates IP3K not only via the GPCR, but also via insulin growth factor-1 (IGF-1R) [100]. Caromile et al. were able to show increased IGF-1R abundance, activated IP3K and repressed MAPK in PSMA-expressing TRAMP mice [101]. While MAPK upregulation has been associated with survival in PCA, the IP3K–AKT–mTOR pathway is increasingly relevant to the progression of multiple cancer types [97,102,103]. According to Caromile and colleagues, PSMA features differential switch-like regulation of these two important pathways via the IGF-1R, which has also been shown to apply to breast cancer [104]. Antibody-mediated cross-linking PSMA, however, induces the ERK/MAPK pathway and has been shown to provide an advantage for proliferation in vitro [105]. Therapeutic targeting of PSMA might therefore induce this switch towards the ERK/MAPK pathway, which is nonetheless oncogenic, albeit weaker in its downstream effects on cancer cell escape mechanisms [100].

Taken together, the PSMA molecule presents with a multitude of functions and seems to be involved in more than one important pathway during cancer cell metabolism and signaling. On one side, the natural function of PSMA to nourish the cell with folate and ensure proper prostate organ function accelerates with higher PSMA expression, thus fueling cancer cell growth, proliferation and migration, as well as tumor angiogenesis. On the other side, PSMA expression putatively reaches a level at which it positively influences cancer cell aggressiveness by interfering with regulation (PTEN) and pathway switches, as seen for MAPK/ERK vs. IP3K–AKT–mTOR. As the cherry on the cake, PSMA even combines these two branches, as Kaittanis et al. showed us, by using free glutamate from the folate metabolism to activate the IP3K–AKT–mTOR pathway via stimulation of GPCRs. These interactions are depicted schematically in Figure 1.

### 3.5. Sequence of CRPC Development: A Putative Scenario of PSMA Detection and Targeting

**Figure 2:**
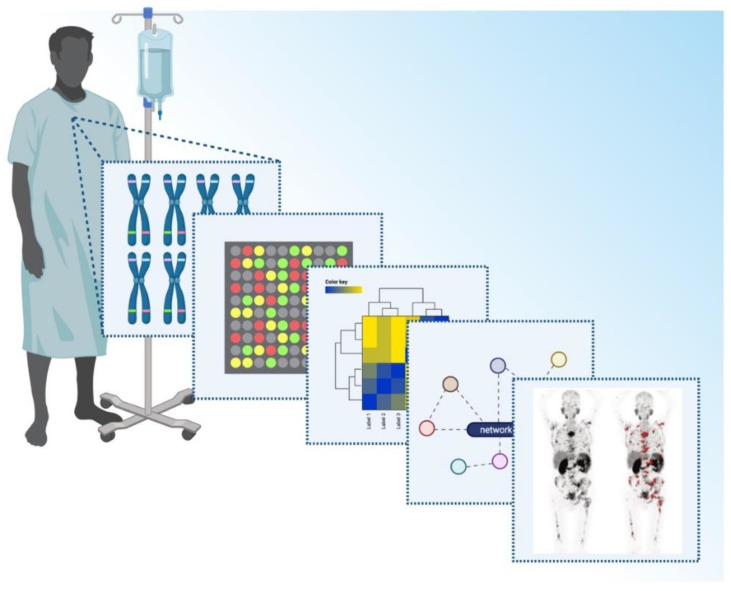
Precision medicine-mediated patient profiling for mCRPC management. Besides routine diagnostics, multiple screenings should be included in the diagnostic process. Genomic analysis for mutation status such as BRCA1/2, PTEN, AR and others should be combined with gene expression analysis and subsequent analysis of interactions and networks. ^68^Ga-PSMA-PET/CT is an indispensable component of this screening process and allows detection of tumor distribution and load. mCRPC: metastatic castration-resistant prostate cancer; BRCA1/2: breast cancer 1/2 gene; PTEN: phosphatase and tensin homolog on chromosome ten; AR: androgen receptor; 68Ga: 68Gallium; PSMA: prostate-specific membrane antigen; PET/CT: positron-emission tomography/computer tomography. This figure was generated using biorender, www.biorender.com, accessed on 8 July 2021.

As described above, PSMA is involved in a multitude of cell metabolism and tumor-aggravating pathways during prostate cancer progression. However, it is also expressed in benign prostate tissue and a variety of other non-malignant conditions [52,106]. In advanced disease, PSMA expression positively correlates with high serum PSA and can be used as a marker for biochemical recurrence and progression, unless it is an immediate effect of ADT [15,88,107]. We therefore may speculate that patients experience a constant rise in PSMA expression levels with progressing disease. There might be a drop in measurable PSMA expression levels due to transient therapy success induced by chemotherapy, for example. ADT, however, induces increased PSMA expression and may therefore promote progression, even if at first regression of the tumor can be observed in most cases. Currently, rising PSMA expression could be used for staging and for temporal and spatial monitoring of the tumor and its metastases, as well as in organs at risk, as described earlier. ^68^Ga-PSMA-PET imaging allows the detection of the PSMA expression level at different sites of disease progression. Drug-related changes in PSMA expression intensity have to be incorporated as described earlier. Additionally, PSMA imaging enables the detection of tumor mass and heterogeneity and together with FDG-PET it might enable proper staging and the early recognition of tumor dedifferentiation to a more aggressive stage [108]. However, it has also been shown that neuroendocrine differentiation is not always associated with increased FDG uptake, indicating that decreasing PSMA expression is not generally replaced by rising GLUT levels and subsequent detectability by ^18^F-FDG-PET [109]. GLUT1 expression, however, seems to bear biomarker characteristics, as it has been shown to correlate with FDG uptake and poor prognosis in high-risk PCA [110]. Based on this, 18F-FDG-PET might serve as a tool to discriminate if patients are eligible for PSMA-RLT or should be treated otherwise, such as with cabazitaxel chemotherapy or agents suitable for NED [111,112]. Although the increase of genes related to ^18^F-FDG-uptake has been found to associate with higher glucose metabolism and PSMA suppression, this requires further investigation for complete understanding of these interactions [68,113]. It has further been shown very recently by Michael Hofman and his colleagues that PSMA-PET-CT is more than suitable for staging even before curative treatment and surgery in patients with high-risk tumors [47]. Not only has PET imaging has been shown to be highly accurate in terms of specificity and sensitivity, but immunohistochemistry of early biopsies and prostatectomies has also shown a correlation with disease stage for morphological classification [23]. In the respective study, Bravaccini et al. presented the highly significant and sensitive performance of PSMA as a histopathological marker for disease staging and the discrimination between low- and high-risk, as well as a positive correlation with the Gleason score. The studies by Hofman et al. as well as by Bravaccini et al. imply that PSMA is merely a marker for advanced disease, nor a target left to heavily treated patients with refractory disease. PSMA bares the potential to be used as both a biomarker and target during early detection of PCA, as well as for monitoring and treatment over the course of the disease. Including molecular parameters such as PSMA gene and protein expression in the grading of early tissue samples represents one more step towards safe and standardized diagnosis by conserving patient-specific features of the disease at the same time. For a schematic representation of the diagnostic process, please refer to Figure 2.

Currently, ^177^Lu-PSMA-617-RLT finds application in patient treatment only after therapy failure and the lack of more treatment options until its approval. The first phase III VISION trial was accomplished, meeting both primary endpoints of overall survival and radiographic progression-free survival [20,114]. After approval, it is intended to make ^177^Lu-PSMA-617-RLT an accessible targeted treatment for >80% of mCRPC patients [19]. Those patients, usually with advanced mCRPC and intense treatment history, benefit from 177Lu-PSMA-617-RLT due to low toxicity and mild side effects, such as nausea, fatigue and dry mouth. Only a few cases displayed adverse events that required reduction, interruption, discontinuation or death related to the drug during the VISION trial. However, the therapy efficiency is described with up to 30% of all patients and a prolongation of overall survival of 15.3 vs. 11.3 months [20]. Although these numbers are robust and promising, it might be discussed whether PSMA-RLT efficiency is hampered by its current late application. Patients that are eligible for PSMA-RLT not only present with advanced disease but also very often with high age and poor general health status. As we showed recently in such advanced heavily treated disease stages, there might be underlying molecular mechanisms of tumor dedifferentiation towards neuroendocrine phenotypes that have not yet reached a measurable stage using the common biomarkers, but may escape adenocarcinoma-focused treatment [115]. Thus, targeting PSMA with radioligand therapy might only lead to minor, if at all, delay of disease progression and not significantly prolong survival nor ameliorate the patient’s wellbeing in those cases. Further, patients with advanced disease often present with a heavy treatment history and multiple metastases, of which visceral metastases have been proven a parameter for poor prognosis [116]. With regard to an earlier application of PSMA-RLT, such as initial presentation with high PSMA and advanced disease, the results of the LuTectomy study will be of great interest. Application of PSMA-RLT before prostatectomy and pelvic nodal dissection to reduce tumor burden to increase surgical safety, and favoring a biochemical response before surgery, seem reasonable and promising [117].

There is a rising number of studies investigating the options of combining PSMA-targeted therapies with other potent systemic or local treatments. We, for example, have been able to show that patients with visceral hepatic metastases undergoing PSMA-RLT can be treated with local SIRT efficiently and that this significantly prolongs survival [118]. This approach bears the option to treat local tumor metastases, even if they present with heterogeneous PSMA expression. In our clinics, we were recently able to show that heterogeneous PSMA expression and patients with low PSMA do not benefit from PSMA-RLT as much as it were desirable [119]. The putatively ongoing dedifferentiation and activation of additional oncogenic pathways in those patients require different treatment options and imaging. Including somatostatin receptor-targeted 68Ga-DOTATATE-PET into the panel seems, therefore, more than reasonable in PSMA low or heterogeneous disease [120].

Although this might be an approach for later and aggressive stages as well as for spontaneous extension of therapies, whenever needed, it represents nonetheless an emergency treatment, as progress had already taken place. One option is the simultaneous use of ADT and PSMA-targeted treatment from the moment the PSMA expression level rises and is detected. This might be at a much earlier timepoint than PSMA imaging and RLT are currently considered. Yet, due to the underlying molecular mechanisms in which PSMA is involved, it might strongly interfere with progression, especially when PTEN is mutated or lost or in the case of BRCA1 and 2 aberrations [121]. It has been shown multiple times by now that PSMA expression correlates positively with disease progression, severity and shortened lifespan [22]. Ergo, it might be very likely that early and combined targeting of PSMA and ADT evolves as a very potent go-to treatment; even more so, as PSMA expression is suppressed during progression to NED, and this therefore comes with loss of a very valuable treatment target, marker for staging and the transition into a more aggressive stage, which is also more difficult to treat. The sequence of PSMA expression during tumor progression putatively begins early and is intensified as the tumor progresses. While in progressing PCA, PSMA expression increases with Gleason score and is associated with recurring serum PSA levels; progression into neuroendocrine prostate cancer (NEPC) is accompanied by decreased PSMA and increased levels of neuroendocrine genes such as FOXA1, ENO2 and SYP [68]. The authors themselves were able to show that SYP and ENO2 gene expression increases in late-stage mCRPC and may point at developing NED, while PSMA gene expression remained stable at first [115]. An early rise in PSMA levels provides an additional anchor for treatments, which should be used as early as possible according to patient indication. ADT, besides being efficiently used to fight tumor progression, can also be used to increase PSMA expression for subsequent therapy, as has been shown for enzalutamide and AA. Heterogeneous expression or decreasing levels might be a sign of tumor progression or dedifferentiation and certainly requires making use of more treatment options, such as local or systemic therapies that have been shown to be well compatible with ADT and PSMA-RLT. Therefore, it may be likely that a decrease in PSMA expression, which can be considered an obligation for progression from castration resistance to NED, can be predicted on the gene expression level of NED-related genes in advanced mCRPC. This might lead to the assumption that adenocarcinoma-like PCA cells eventually dedifferentiate from mCRPC into a more aggressive stage, and that this process is transiently allowed by first rising and then decreasing PSMA expression.

## 4. Theranostics: PSMA as a Target for Imaging and Treatment

The principle of theranostics combines the features of a biomarker that is eligible for both targeting and detection. This enables a highly patient- and disease-stage-specific monitoring of biomarker distribution and abundance, as well as direct targeting of the marker-expressing sites with few side effects. As PSMA expression on tumor cells correlates with disease progression, its detection by labeling allows close to real-time detection of tumor growth and migration [62,122]. Regrettably, ^68^Ga-PSMA-PET is used rarely for this purpose, but only to determine eligibility for ^177^Lu-PSMA-617 therapy after complete failure and refractory PCA in daily clinical business. This might, however, more be an issue of feasibility and financial support by compulsory health insurance funding than of clinical justification, which might be an international issue. Even though PET imaging is a complex procedure, which wants to be planned and conducted within a certain time frame and requires the patient’s compliance and consent, it is yet less invasive than taking a biopsy. PSMA can also be detected in circulating tumor cells, which does not require more than an additional blood sample from the patient, also called liquid biopsy. A general rise in PSMA expression can therefore be easily detected from this blood sample. However, although it has been shown that PSMA can be detected in CTCs and additionally correlates with disease, this does not localize the tumor’s distribution nor allow grading of local re- or progression, heterogeneity or success of localized therapies [123,124]. An intelligent biomarker profile for PCA disease staging, including PSMA, would surely be of great advantage, but it might not replace state-of-the-art PSMA-targeted imaging, rather than accomplish the process of profiling [22]. With regard to this, liquid biopsies could be used for tight PSMA expression detection during treatment and surveillance [125]. This might accomplish the commonly used PSA test and response, which is less indicative in advanced stages such as mCRPC [126]. In the case of a stronger PSMA level increase, ^68^Ga-PSMA-PET should be included in the diagnostic panel more often and earlier according to the rate of progression and depending on the treatment strategy to improve staging [24]. Further, independent of PSMA, global molecular analyses of direct patient material are desperately needed to fully understand underlying interactions and mechanisms, as in vitro studies do not always reflect in vivo situations [127].

The feature of binding a ligand as a receptor and internalizing it paved the way to use PSMA as a specific ferry to transfer antibodies, small molecules bound to radionuclides directly into the tumor. Although antibodies were the first approved imaging agents and seem to not be toxic on the salivary glands, they require longer clearance and seem to have higher hematologic toxicity, as well as sensitivity and specificity issues [62]. As PSMA targeting further evolved, low-molecular-weight ligands based on the glutamate–urea cargo-carrying derivatives demonstrated excellent imaging and tumor targeting [128]. In Germany, ^68^Ga-DOTATATE-PSMA and ^177^Lu-DOTATATE-PSMA-617 are extensively used for imaging and radioligand therapy, respectively. A 12-center study from Germany with 145 enrolled patients revealed a biochemical response, determined by a ≥50% decline in serum PSA in >45% of the patients [129]. Compared to this, the VISION trial was closed very recently with promising results, leading to impending approval by the FDA [19,114]. This review is not thought to discuss the multitude of ligands and optional radionuclides such as ^225^Ac which exhibit unfavorable side effects, such as unspecific binding and significantly higher toxicity [130]. While there might surely be indications for their application ^177^Lu seems to be the radionuclide of choice and 177Lu-DOTATATE-PSMA-617 the soon-to-be only approved PSMA-RLT [131]. While, currently, PSMA-RLT is the last therapy option for progressing PCA, its earlier application would be desirable, based on the patient’s eligibility. To meet all requirements for this therapy, patients should be selected on the recommendations of the prostate cancer trials working group 3 (PCWG 3) and by an interdisciplinary team of urologists, oncologists and nuclear medicine physicians [10,131]. 68GA-PSMA-PET imaging should definitely be included into this panel; however, cutoffs for PSMA-RLT success are vaguely defined. ^68^Ga-PSMA-11 uptake of >1.5 times greater than normal liver uptake was determined as predictive for therapy response by Michael Hofman and his colleagues [112]. While a standard cutoff has yet to be defined, it has been shown that PSMA-RLT efficacy positively correlates with the intensity of tumor uptake [132]. There is no question that there is a lot of work that still needs to be carried out in terms of patient profiling and uptake cutoff determination to include PSMA-RLT more flexibly into mCRPC treatment sequences. However, PSMA-PET imaging should be used more widely for staging at initial presentation, before curative surgery or radiotherapy, because it has been shown to be superior to combined CT and bone scanning [47].

## 5. Combination of Novel Agents with PSMA-RLT and Management of mCRPC

Covering all promising treatment strategies under investigation for all stages of PCA is out of the scope of this review. As PSMA is most relevant to mCRPC, and to meet the scope of this review, we will focus on treatments that target this stage specifically. Further, only approved or close to-approval treatments will be discussed.

The stage of castration resistance is characterized by lack of sensitivity to androgen deprivation, rising PSMA expression and the accumulation of mutations in DNA damage repair (DDR) genes such as BRCA1/2. Dysregulation in DDR pathways leads to mismatch mutations, aberrant gene expression and cancer cells escaping apoptosis. Therefore, it seems reasonable to target these pathways in a combinatorial, consecutive or alternating treatment sequence with novel and potent agents.

The most obvious and best investigated to date is the combination of PSMA-RLT with novel ADTs such as abiraterone acetate (AA) and enzalutamide. Although the outcome of the VISION trial should be taken into consideration, before application outside compassionate use, this option seems more than realistic for future application [19,20]. The toxicity profile of both therapies is favorable and heavy side effects should not be expected [133]. ADT with AA or enzalutamide will at first suppress the androgen receptor signaling axis and thus cancer cell growth. Secondly, AR inhibition leads to increased PSMA expression and therefore to the generation of the target for the second treatment: ^177^Lu-PSMA-617-RLT. PSMA-RLT functions at two sites: binding of PSMA leads to internalization and suppresses the enzymatic function of PSMA. This leads to less folate uptake and less free glutamates that in turn might activate downstream pathways such as IP3K–AKT–mTOR signaling. Further, the radionuclide, in this case ^177^Lu, that is internalized with the PSMA receptor induces double-strand breaks and downstream apoptosis of the tumor cells. The combination of ADT and PSMA-RLT therefore offers to prevent or at least delay disease progression and the development of resistance to agents such as AA and enzalutamide. By interrupting the downstream effects on tumor cell proliferation and growth via enhanced PSMA expression, this treatment combination interferes with the accumulation of gain-of-function point mutations in the AR gene and mismatch repair.

Intact DDR mechanisms mediated by the BRCA2 gene might hamper the success of induced double-strand breaks, by simply maintaining genomic stability in the tumor cell. Mutations of the BRCA1/2 gene are associated with a higher risk of certain tumors, including prostate cancer. Moreover, BRCA mutations are a strong prognosticator of response to poly-ADP ribose polymerase inhibitors (PARPi) such as olaparib and platinum [134,135]. The application of PARPi requires a preliminary screening and determination of the genomic status, as certain aberrations such as BRCA1/2 mutations have been shown to be predictive for the outcome. Olaparib, an approved PARPi, however, has also been shown to be effective with a non-BRCA1/2 mutated profile, so that its application is not restricted regarding this [136]. It has further been shown that the AR is required for the maintenance of homologous recombination (HR). AR gene aberration and mutation might therefore interfere with and functionally impair HR and PARP pathways, which are upregulated in prostate cancer cells. Tumors with HR defects have been shown to become sensitive to PARPi and a combination with ADT showed promising results in high-risk mCRPC [32,137]. In a scenario of early castration resistance and the optional presence of BRCA mutations, a consecutive treatment with ADT, followed by olaparib, followed by ADT is conceivable [32,137,138]. Including PSMA-RLT into this sequence seems realistic as induction of more double-strand breaks and less repair might lead to more tumor cell death and thus to a more successful treatment. A study to investigate the combined use of PSMA-RLT and olaparib is currently being conducted at the Peter MacCallum Cancer Center, Australia and its results are curiously awaited [139]. This has similarly been described for the use of platinum and likewise seems a reasonable approach [140]. In terms of PSMA-RLT alone, one might meet the limitations of this therapy in patients with upregulated PARP and BRCA2 mutations and thus DNA repair despite accumulated double-strand breaks. This might explain the roughly 30% of non-responders despite intense PSMA expression [141], although there might be more complex interactions, as we were able to show that non-responders must be discriminated from late responders [142]. Further, undetected dedifferentiation and progression shall also be taken in account [143]. A combination of PSMA targeting with additional immunotherapy, such as with pembrolizumab, has also been shown to be effective; however, it is currently not approved for mCRPC in the EU [144]. Early application of PARPi is worth taking a look at, because PARP overexpression seems to not only promote DDR, but also enhance AR transcriptional function in advanced mCRPC and thus prevent cancer cell apoptosis [138].

These studies and data are very promising; however, none of these treatments should be applied blind and without awareness of the patient’s gene status. Therefore, genomic profiling and gene expression analyses should be considered an important part of routine management of mCRPC patients for true precision medicine [45]. Further, more prospective studies on combined treatments in mCRPC are desperately needed. As ADT as well as olaparib are already approved treatments and the approval of PSMA-RLT is in the offing, new opportunities for mCRPC treatment are under way. This will definitely broaden the spectrum of therapies to choose from, as well as promising a more successful treatment of the currently incurable stage of mCRPC. Due to its low toxicity and usual tolerability, it is unlikely that PSMA-RLT would interfere and prevent the efficacy other any other treatment. If applied in decent temporal sequences as a consecutive or alternating agent, there is no reason to assume heavy side effects. With regard to this, PSMA-RLT could also be applied in combination with other non-cytotoxic therapies.

## 6. Conclusions

Metastatic CRPC is a complex disease and during its progression tumor cells activate multiple pathways to escape common selective mechanisms such as senescence and apoptosis. The increased expression of PSMA is part of cancer cell survival strategies and the activation of oncogenic pathways. There is a huge need for accurate profiling on the basis of the molecular interactions. This could be met by using global genomic and gene expression profiling of patient material. Biopsies, once taken, should be subjected to global screenings for suspected key players in defining disease severity and enable a precise stage classification. Additionally, circulating tumor cells as well as peripheral blood should be used to detect biomarker expression and reveal correlations with progression. Indispensable for this ultimate patient screening is imaging, whether it may be global imaging tracers, such as FDG to quantify tumor mass and identify metastases, or specific ligands that allow stage-specific expression, such as PSMA. PSMA and SSRs are definitely targets that allow detection of tumor heterogeneity and prediction of progression and therapy efficacy. As there are multiple ways for tumors to progress and inter-patient fluctuations in marker expression, clinicians need a precise profile of each individual patient. Besides genome and transcriptome multi-omics, screening approaches should be taken into account. Current approved and impending treatment options provide powerful tools to fight the uncurable stage of mCRPC. Yet, potent treatments such as PSMA-RLT, once approved, and PARPi should be considered for earlier use, and our current understanding of rigid treatment lines should be discussed. Combinatorial, consecutive and alternating treatment strategies fitted to the patient’s disease profile are the future of mCRPC treatment.

## Data Availability

This study did not include any data.
